# Fiscal policy for stabilization during the COVID-19 crisis: the role of social spending

**DOI:** 10.1080/13504851.2023.2259583

**Published:** 2023-09-15

**Authors:** Philipp Heimberger

**Affiliations:** Vienna Institute for International Economic Studies (Wiiw), Vienna, Austria

**Keywords:** Fiscal policy, Covid-19 crisis, financial crisis, automatic stabilizers, social spending, E62, H11, H61

## Abstract

This paper analyses whether government spending for social protection contributed more to counteracting the COVID-19 crisis than in previous episodes. Based on data for 27 EU countries over 1995–2021, we find that social spending is countercyclical and contributed to stabilization during the pandemic. However, we do not find evidence that overall social spending during 2020–2021 was more countercyclical then before. While unemployment spending played a particularly strong countercyclical role during the pandemic years 2020–2021, the cyclical behaviour of spending on old age, family and children, and sickness and disability was more in line with the past. The cyclicality of unemployment spending differs when comparing Continental with Southern, Nordic and Eastern EU countries.

## I. Introduction

The question whether and how fiscal policy contributed to stabilizing the economy during the COVID-19 crisis has attracted the interest of researchers and policymakers (e.g. Carvalho, Cardomingo, and Toneto 2021; Deb et al. 2021; Gourinchas et al. 2021). Fiscal policy in advanced economies was significantly countercyclical during the pandemic years 2020 and 2021; this implies that fiscal policy generally counteracted the economic downturn, although the cyclical behaviour of fiscal policy differed across countries (Heimberger 2023). However, the literature has so far focused on overall fiscal policy and its cyclical properties. This paper studies whether different components of government spending for social protection (henceforth: social spending) were more countercyclical during the pandemic than in the past. Existing studies do not always agree on the cyclical behaviour of social spending, as they use different data in terms of country and time coverage (Abbott and Jones 2012; Afonso and Jalles 2013; Arze Del Granado, Gupta, and Hajdenberg 2013, D’Addio 2015; Ayala-Canon, Delgado-Rodriguez, and Lucas-Santos 2022; Darby and Melitz 2008; Galeano et al. 2021). This paper presents the first analysis including data for the pandemic years 2020 and 2021 by focussing on EU member states.

## II. Data and econometric approach

We use Eurostat data on general government expenditure by function (COFOG) to measure government spending for social protection in 27 EU countries over the time period 1995–2021. The data allow us to distinguish the subcomponents sickness and disability, old age, family and children, unemployment, and housing.^1^^1^Notably, overall social spending also includes spending on survivors and other small components, which we do not consider in this paper as they are less important. We separately estimate the cyclical behaviour of these components of social spending:

(1) $$\begin{array}{cc} & \mathrm{SOCEX}P_{i,t}=c+\mathrm{\alpha CYCL}E_{i,t}+\mathrm{\beta ELE}C_{i,t}+\\ & \mathrm{\gamma PDEB}T_{i,t-1}+\mathrm{\delta COVI}D_{i,t}+\\ & \mathrm{\eta CYCL}E_{i,t}*\mathrm{COVI}D_{i,t}+\tau_{i}+\varepsilon_{i,t} \end{array}$$

where $\mathrm{SOCEX}P_{i,t}$ refers to government expenditure on social protection in % of potential output in country i at time t in the different categories. c is a constant. $\mathrm{Cycl}e_{i,t}$ measures cyclical conditions, proxied by the output gap. $\mathrm{ELE}C_{i,t}$ is a dummy variable set to 1 in each federal election year in order to test whether social spending is more or less expansionary during election years. $\mathrm{PDEB}T_{i,t-1}$ is the first lag of the public-debt-to-GDP ratio, which allows us to test whether higher initial public debt levels affect social spending. $\tau_{i}$ captures country-fixed effects. $\varepsilon_{i,t}$ is the error term. To test whether the COVID-19 crisis affected the cyclicality of social spending, we include an interaction term of the variable capturing cyclical conditions with a dummy variable for the COVID-19 crisis years 2020–2021 ($\mathrm{CYCL}E_{i,t}*\mathrm{COVI}D_{i,t}$).

The output gap captures the difference between actual output and potential output (in % of potential output), indicating the cyclical position of an economy. Endogeneity is an important issue: the error term is likely to be positively correlated with the output gap if exogenous fiscal shocks affect both social spending and economic activity. We address this by running a regression of the social spending variable on a component of the output gap unaffected by exogenous discretionary fiscal shocks (e.g. Gali and Perotti 2003). We use an instrumental variable approach, where our instruments for the output gap are the lag of the country’s own output gap and the contemporaneous value of the US output gap. This is based on the assumption that the US output gap does not respond contemporaneously to cyclical developments in other countries, while there is such an impact of US developments on other countries (Fatas and Mihov 2010). Our methodology builds on Heimberger (2023) who uses the same IV approach. However, we go beyond Heimberger (2023), who studies the cyclicality of overall fiscal policy during the COVID-19 crisis, by zooming in on the cyclical behaviour of social spending and its subcategories. As a robustness check, we also look at results from a dynamic model estimated by using GMM, where we instrument the social spending variable and the output gap with their own lags (Blundell and Bond 1998).

If social spending is countercyclical, the output gap has a negative sign, implying that an increase in the (expected) output gap is related to a fall in social spending. Vice versa, a positive coefficient estimate would point to procyclicality.

We downloaded data on output gaps and public-debt-to-GDP from the AMECO (November 2022) database. We obtained data on elections from various election data sources, including the website electionresources.org.

## III. Results

Table 1 shows the instrumental variable two-stage least squares (IV2SLS) results. The instruments are highly correlated with the instrumented variable. Weak instrument tests reject that the instruments are weak; hence, we move forward with the assumption that the instrumental variable approach is sufficiently strong. The coefficient estimates on elections are typically insignificant. Higher initial public debt levels are related to higher social spending, in particular higher old age spending. A major result is that social spending is, overall, countercyclical, as indicated by the negative and significant output gap coefficient. The coefficient estimate for the interaction term of the COVID-19 dummy with the output gap, however, suggests that we cannot reject that the cyclicality of social spending during the pandemic was not different from the past. When we test the cyclical behaviour of different components of social spending, we do not find significant interaction terms for sickness and disability, old age, family and children, and housing. This suggests that the cyclicality of spending of these components during the pandemic was statistically indistinguishable from the past. Our finding that old age spending is overall countercyclical is consistent with earlier evidence reported in Darby and Melitz (2008). Importantly, we find that unemployment spending was more countercyclical during the pandemic. Notably, several EU countries (temporarily) expanded and extended unemployment benefits during the COVID-19 crisis to shield workers from losses due to the labour market consequences of the pandemic (IMF 2021).

**Table 1.** Panel regression results (IV2SLS), 27 EU countries over 1995–2021.

	*Dependent variable:*					
	(1)	(2)	(3)	(4)	(5)	(6)
	SOCIAL	SICK	OLD	FAMI	UNEM	HOUS
OG	−0.110***	−0.0003	−0.081***	−0.007	−0.023*	−0.001
	(0.035)	(0.012)	(0.025)	(0.008)	(0.013)	(0.003)
COVID	0.415	0.020	0.503***	0.228**	−0.091	−0.017
	(0.337)	(0.135)	(0.185)	(0.104)	(0.108)	(0.030)
PDEBT_*t*–1_	0.026***	0.0001	0.021**	0.001	0.002	−0.001*
	(0.008)	(0.002)	(0.010)	(0.002)	(0.002)	(0.001)
ELEC	0.059	0.002	0.077**	0.013	−0.016	0.002
	(0.050)	(0.012)	(0.035)	(0.014)	(0.016)	(0.006)
OG*COVID	−0.047	0.004	0.023	0.021	−0.035**	−0.004
	(0.079)	(0.021)	(0.045)	(0.014)	(0.017)	(0.006)
Constant	18.804***	1.872***	10.703***	2.470***	1.147***	0.221***
	(0.591)	(0.148)	(0.715)	(0.137)	(0.175)	(0.059)
Observations	703	634	635	635	635	635
R^2^	0.905	0.853	0.876	0.891	0.846	0.829
Adjusted R^2^	0.900	0.846	0.869	0.886	0.838	0.821

Note: *p<0.1; **p<0.05; ***p<0.01. SOCIAL… social spending; SICK… spending on sickness and disability; OLD… spending on old age; FAMI… spending on family and children; UNEM… spending on unemployment; HOUS… spending on housing; OG… output gap; PDEBT… public-debt-to-GDP ratio; ELEC… elections; COVID… COVID-19 crisis dummy. All government spending variables entered in % of potential output. Country-fixed effects are included in all models, but not shown for brevity. Standard errors (clustered at the country-level) are given in parentheses. *p<0.1; **p<0.05; ***p<0.01.

Table 2 reports additional results on the cyclical behaviour of unemployment spending during the pandemic. First, we include a lagged unemployment spending variable as an additional regressor. We again find that unemployment spending was, on average, more counter-cyclical in 2020/2021. Second, we test the robustness of our IV2SLS results by using a GMM estimator. IV results are potentially biased when we include country-fixed effects and the lagged dependent variable simultaneously. However, re-estimating the models using GMM yields very similar results. We use system-GMM, which is potentially less affected by the weak instrument problem than difference-GMM; prior studies point to the preference of system-GMM with fiscal reaction functions (e.g. Bernoth, Hughes Hallett, and Lewis 2015). Therefore, we provide results based on a one-step system-GMM approach (Blundell and Bond 1998), where we use the *t*-2 and *t*-3 lags of the social spending variable and the output gap as instruments. The GMM results confirm our findings. Furthermore, we extend our analysis by considering different country groups: we split our sample into Continental, Nordic, Southern and Eastern EU countries.^2^^2^Continental: Austria, Belgium, France, Germany, Netherlands, Luxembourg. Nordic: Denmark, Finland, Sweden. Southern: Greece, Italy, Cyprus, Portugal, Spain, Malta. Eastern: Czechia, Bulgaria, Estonia, Croatia, Latvia, Lithuania, Hungary, Poland, Romania, Slovenia, Slovakia. Our findings suggest that the cyclical behaviour of social spending differs across country groups: in the Continental EU countries, we find significantly stronger countercyclicality of unemployment spending during the pandemic. However, we do not find significant interaction terms for Nordic, Southern and Eastern EU countries, respectively.

**Table 2.** Further results on whether unemployment spending was more countercyclical during the COVID-19 crisis.

	OG*COVID
*IV2SLS as in*Table 1*(all 27 EU countries)*	
	−0.035**
*IV2SLS with lagged dependent variable (all 27 EU countries)*	
	−0.094***
*System-GMM with lagged dependent variable (all 27 EU countries)*	
	−0.075***
*IV2SLS (Continental EU countries)*	
	−0.087**
*IV2SLS (Nordic EU countries)*	
	−0.127
*IV2SLS (Southern EU countries)*	
	−0.025
*IV2SLS (Eastern EU countries)*	
	−0.004

SOCIAL… social spending; UNEM… spending on unemployment; HOUS… spending on housing; OG… output gap; COVID… COVID-19 crisis dummy. All government spending variables entered in % of potential output. The table just shows the coefficient estimates and significance levels of the interaction term between output gap and COVID-19 dummy (OG*COVID). All other control variables as specified in Equation (1) were also included in the model estimations, but results are not shown for brevity. *p<0.1; **p<0.05; ***p<0.01.

## IV. Conclusions

This paper has analysed the cyclical behaviour of government spending for social protection. We find that social spending is, overall, countercyclical and contributed to counteracting the pandemic downturn. However, we fail to find evidence that overall social spending during 2020/2021 was more countercyclical than before. Our findings suggest that unemployment spending played a particularly strong countercyclical role during the pandemic years, but the cyclicality of spending on old age, family and children, sickness and disability, and housing during the COVID-19 crisis cannot be statistically distinguished from earlier periods. We find that unemployment spending was more countercyclical during the pandemic in Continental EU countries, but not in Southern, Nordic and Eastern EU countries. This finding is consistent with the literature showing that continental European countries tend to have the most forceful unemployment benefit systems (e.g. Boeri 2011). Future research could assess the cyclical behaviour of government spending on health by using Eurostat’s COFOG data. Further research is needed to test whether the cyclical behaviour of social spending during the COVID-19 crisis in non-EU advanced economies or emerging-market economies differed from those in EU countries.
